# Scopolin Attenuates Osteoporotic Bone Loss in Ovariectomized Mice

**DOI:** 10.3390/nu12113565

**Published:** 2020-11-20

**Authors:** Eunkuk Park, Jeonghyun Kim, Hyun-Seok Jin, Chun Whan Choi, Tae Hyun Choi, Sangho Choi, Dam Huh, Seon-Yong Jeong

**Affiliations:** 1 Department of Medical Genetics, Ajou University School of Medicine, Suwon 16499, Korea; jude0815@hotmail.com (E.P.); danbi37kjh@hanmail.net (J.K.); 2 Department of Biomedical Sciences, Ajou University Graduate School of Medicine, Suwon 16499, Korea; 3 Department of Biomedical Laboratory Science, College of Life and Health Sciences, Hoseo University, Asan 31499, Korea; microchin@hanmail.net; 4 Natural Products Research Institute, Gyeonggi Institute of Science & Technology Promotion, Suwon 16229, Korea; cwchoi78@gmail.com; 5 Department of Molecular Imaging, Korea Institute of Radiological and Medical Sciences, Seoul 01812, Korea; thchoi@kirams.re.kr; 6 International Biological Material Research Center, Korea Research Institute of Bioscience and Biotechnology, Daejeon 34141, Korea; decoy0@kribb.re.kr; 7 Dongwoodang Pharmacy Co. Ltd., Yeongchen 38819, Korea; herbleader@omniherb.com

**Keywords:** *Lycii radicis cortex*, scopolin, OVX mice, osteoporosis, osteoclast, osteoblast

## Abstract

Bone remodeling is a renewal process regulated by bone synthesis (osteoblasts) and bone destruction (osteoclasts). A previous study demonstrated that *Lycii radicis* cortex (LRC) extract inhibited ovariectomized (OVX)-induced bone loss in mice. This study investigated the anti-osteoporotic effects of bioactive constituent(s) from the LRC extract. The effective compound(s) were screened, and a single compound, scopolin, which acts as a phytoalexin, was chosen as a candidate component. Scopolin treatment enhanced alkaline phosphatase activity and increased mineralized nodule formation in MC3T3-E1 pre-osteoblastic cells. However, osteoclast differentiation in primary-cultured monocytes was reduced by treatment with scopolin. Consistently, scopolin treatment increased osteoblast differentiation in the co-culture of monocytes (osteoclasts) and MC3T3-E1 (osteoblast) cells. Scopolin treatment prevented bone mineral density loss in OVX-induced osteoporotic mice. These results suggest that scopolin could be a therapeutic bioactive constituent for the treatment and prevention of osteoporosis.

## 1. Introduction

Bone remodeling during vertebrate development is governed by the coordinated action of bone formation (osteoblasts) and resorption (osteoclasts) [1,2]. Bone formation is initiated by high alkaline phosphatase (ALP) activity and is regulated by mineralization, collagen synthesis, and osteoblastic proliferation and differentiation [3]. However, bone resorption is associated with osteoclastic proliferation and tartrate-resistant acid phosphatase (TRAP) activity [4]. Old or damaged bone tissue is removed by the action of osteoclasts on the bone surface, followed by the formation of new bone tissue by osteoblasts [1,5]. Imbalanced regulation of the two bone remodeling sub-processes results in many metabolic bone diseases, such as osteoporosis [6]. Osteoporosis is a progressive bone disease characterized by excessive bone resorption, decreased bone mass and density, and associated increased risk of bone fragility and susceptibility to fracture [7]. Osteoporosis is the most common disease in the elderly, particularly in postmenopausal women, with an estimated 100 million people suffering from debilitating bone diseases globally [8,9]. Numerous pharmacological medications for the treatment of osteoporosis have been discovered; however, some of them show negative effects with long-term treatment, including stomach upset and high risk of breast and endometrial cancers [10,11].

During the past decade, traditional herbal plants have been used for medicinal purposes because they are more suitable for long-term treatment with fewer side effects than pharmacological medications. Herbal medicines are widely used to improve bone health and as alternative therapeutic options for osteoporosis [12]. Many researchers have identified and modified bioactive ingredients from plants as herbal medicines [13,14]. *Lycii radicis* cortex (LRC), the root bark of *Lycium chinense*, is broadly regarded as a traditional medicine in eastern Asia [15]. Physiologically bioactive compounds isolated from LRC extract include apigenin, luteolin, kaempferol, quercetin, oleanolic acid, and ursolic acid [15,16]. Treatment with LRC has been shown to improve insulin resistance and lipid metabolism in type 2 diabetic rats [17]. Studies have demonstrated that LRC extract enhances insulin resistance and secretion in the regulation of glucose lipid metabolism and has a protective effect against hepatic steatosis, leading to increased antioxidant activity and enhanced immune system [17,18]. Our previous study demonstrated the therapeutic effect of LRC extract on bone formation in vitro and in vivo by increasing the proliferation and differentiation of osteoblasts [19]. Despite the beneficial effects of LRC extract on osteoporosis, the specific compound(s) in LRC with bioactive effects on osteoporosis have not been studied.

Here, we explored the therapeutic possibilities of a single anti-osteoporotic compound (scopolin) isolated from LRC extract. We evaluated the bone formation-enhancing effects of scopolin on osteoblastic MC3T3-E1 cells. In addition, we examined bone formation in a monocyte/MC3T3-E1co-culture system to characterize the differentiation of osteoblast and osteoclast cells. Finally, we investigated the protective effect of scopolin on OVX-induced bone loss in an osteoporotic mouse model.

## 2. Materials and Methods

### 2.1. Fractionation, Isolation, and Structure Elucidation

LRC was obtained from Dongwoodang Pharmacy Co., Ltd. (Yeongcheon, Gyeongsangbuk-do, Korea). Voucher specimens (GL0180), authenticated by Dr. Chun Whan Choi, were deposited at the herbarium of the Bio-center, Gyeonggi Business & Science & Accelerator, Suwon, South Korea. The ethanol extract of LRC (254 g) was evaporated, suspended in H_2_O, and then partitioned successively into CH_2_Cl_2_ (A, 1.6 g), EtOAc (B, 3.6 g), n-BuOH (C, 120.1 g), and aqueous (D, 128.1 g) fractions. Fraction D was chromatographed on a Diaion HP-20 gel (1000 g) column and eluted with a H_2_O-MeOH solvent gradient system (0% MeOH, 35% MeOH, 70% MeOH, and 100% MeOH) to give four fractions (D1-D4). Fraction D2 (14.5 g) was subjected to RP-18 gel (200 g) column chromatography and eluted with H_2_O-MeOH (100:0 to 0:100) to produce seven subfractions (D2-1 to D2-7). Subfraction D2-5 (524 mg) was subjected to preparative HPLC and eluted with MeOH-H_2_O/0.1% formic acid (10:90) to give compound 1 (2.1 mg) (Figure S1). The structure of the isolated compound was determined using ^1^H-NMR, ^13^C-NMR, and mass spectrometry (Figure S2) and compared with reference data [20].

### 2.2. Reagents and Cell Culture

Mouse preosteoblastic MC3T3-E1 cells were obtained from the RIKEN Cell Bank (Tsukuba, Japan) and cultured in α-modified minimal essential (α-MEM) medium (Gibco; Waltham, MA, USA) supplemented with streptomycin (100 μg/mL; Gibco, Grand Island, NY, USA), penicillin (100 U/mL; Gibco, Grand Island, NY, USA), and 10% fetal bovine serum (FBS) (Gibco, Grand Island, NY, USA). Preosteoblast differentiation was stimulated by adding β-glycerophosphate (10 mM) (Sigma-Aldrich; St. Louis, MO, USA) and an osteogenic medium containing ascorbic acid (50 μg/mL) (Sigma-Aldrich; St. Louis, MO, USA) after allowing 24 h for cell adherence (day 0). The cells were incubated in a humidified atmosphere at 37 °C containing 5% CO_2_, and the medium was changed every 3 days.

### 2.3. Water-Soluble Tetrazolium Salt (WST) Assay

The cells were cultured in a 96-well plate overnight and co-treated with different concentrations of scopolin (1, 5, and 10 μM) in the induction medium for 48 h. Cell viability was determined with a D-Plus™ CCK cell viability assay kit (Donginls; Seoul, Republic of Korea). The cells were incubated with water-soluble tetrazolium salt (WST) solution (5 mg/mL in PBS) for 2 h, and their absorbance values were analyzed using a microplate reader (BioTek, Winooski, VT, USA) at 450 nm and 655 nm (reference wavelength).

### 2.4. ALP Activity and Staining

Osteoblast differentiation was measured using ALP activity in total cell lysates in a buffer containing 10 mmol/L of Mg^2+^, 0.5% Triton X-100, 1 mmol/L of Tris-HCl (pH 8.8), and 5 mmol/L of *p*-nitro-phenylphosphate. The absorbance value was measured at 405 nm (BioTek, Seoul, Korea). ALP staining in the differentiated osteoblasts was performed by fixing them with 4% paraformaldehyde (PFA), followed by staining with SIGMA*FAST*^™^ BCIP^®^/NBT (Sigma-Aldrich; St. Louis, MO, USA; B5655). The ALP-positive osteoblast cells were examined under a microscope (KERN & SOHN GmbH; Balingen, Germany, and Korea Lab Tech; Gyeonggi-do, Republic of Korea).

### 2.5. Analysis of Mineralized Nodule Formation in Osteoblast Cells

The MC3T3-E1 cells were incubated in a 48-well plate and treated with three different concentrations of scopolin (1, 5, and 10 μM) for 21 days. Matrix mineralized nodule formation was measured using Alizarin Red S (Sigma-Aldrich; St. Louis, MO, USA) staining. After 21 days of incubation with Scopolin in the osteoblast induction medium, the induction medium was removed and the mineralized osteoblastic cells were washed three times with PBS. The cells were fixed with 70% ethanol for 10 min, and nodule formation was evaluated by staining with 40 mM alizarin red S (Sigma-Aldrich). Positive alizarin red S staining was identified using microscopes (KERN & SOHN GmbH and Korea Lab Tech, Balingen Germany).

### 2.6. In Vitro Generation of Osteoclasts and the Co-Culture System

For the differentiation of osteoclasts, monocytes were separated from mouse bone marrow cells in induction medium containing 1 mM ascorbate-2-phosphate (Sigma-Aldrich; St. Louis, MO, USA). The identification of monocyte cells was confirmed via a monocyte marker (CD11b antibody; BioLegend; San Diego, CA, USA; 101207), using a FACS Aria III cell sorter (BD Biosciences, San Jose, CA, USA) with the FACS Diva software (BD Biosciences; San Jose, CA, USA)). For induction of differentiation to osteoclasts, the monocytes were incubated in the buffer containing 50 ng/mL of receptor activator of nuclear factor-κB (NF-κB) ligand (RANKL) (PeproTech; Cranbury, NJ, USA) and 30 ng/mL macrophage colony-stimulating factor-1 (M-CSF) (PeproTech; Rocky Hill, CT, USA) [21]. The differentiation medium was changed every 2 days. MC3T3-E1 cells (2 × 10^4^ cells/well) and the isolated monocyte cells (4 × 10^4^ cells/well) were co-incubated in media containing β-glycerophosphate (10 mM) and ascorbic acid (50 µg/mL).

### 2.7. TRAP Staining and Activity Assay

After induction of osteoclast differentiation, the cells were fixed with 4% PFA for 10 min and washed three times with PBS. The differentiated osteoclasts were analyzed using TRAP staining and activity assay with an Acid-Phosphatase Kit (Sigma-Aldrich; St. Louis, MO, USA; 387A). TRAP-positive cells, including three or more multinucleated nuclei, were calculated under microscopes (KERN & SOHN GmbH and Korea Lab Tech, Balingen Germany). Osteoclastogenesis-induced monocytes were subjected to TRAP activity assay using TRACP & ALP Assay Kit (Takara Bio Inc.; Shiga, Japan) as described by the manufacturer’s protocol. The absorbance of TRAP activity was analyzed at 405 nm (BioTek, Seoul, Korea) and expressed as a percentage of the untreated control.

### 2.8. Quantitative Reverse-Transcription PCR (qRT-PCR)

Total RNA was extracted from the cultured cells using TRIzol reagent (Invitrogen; Carlsbad, CA, USA) and complementary DNA (cDNA) was produced using a RevertAid™ H Minus First Strand cDNA Synthesis Kit (Fermentas; Hanover, MD, USA; K1622), according to the manufacturer’s recommendations. PCR amplifications were performed using a SYBR Green I qPCR kit (Takara Bio Inc.) at 40 cycles with 150 ng of cDNA using Bio-Rad CFX (Bio-Rad; Hercules, CA, USA). The specific primers were as follows: for mouse *Alpl*, 5′-CCA ACT CTT TTG TGC CAG AGA-3′ and 5′- TGA CAT TCT TGG CTA CAT TGG TG-3′; for mouse *Runx2*, 5′-TAA AGT GAC AGT GGA CGG TCC C-3′ and 5′- TGC GCC CTA AAT CAC TGA GG-3′; for mouse *Bglap*, 5′-TAG TGA ACA GAC TCC GGC GCT A-3′ and 5′-TGT AGG CGG TCT TCA AGC CAT-3′; for mouse *Sp7*, 5′-ATG GCG TCC TCT CTG CTT G-3′ and 5′-TGA AAG GTC AGC GTA TGG CTT-3′; for mouse *Tnfsf1*1, 5′-TGC CTA CAG CAT GGG CTT T-3′ and 5′-AGA GAT GAA CGT GGA GTT ACT-3′; for mouse *Nfatc1*, 5′-GGA GAG TCC GAG AAT CGA GAT-3′ and 5′-TTG CAG CTA GGA AGT ACG TCT-3′; and for mouse *Gapdh*, 5′-AGG TCG GTG TGA ACG GAT TTG -3′ and 5′- TGT AGA CCA TGT AGT TGA GGT CA-3′. Gene expression was quantified by normalizing to *Gapdh* expression using the comparative threshold (Ct) method, as described by the manufacturer (Bio-Rad software). The values were expressed as fold change compared to the control. The relative gene expression was calculated as 2^−ΔCt^ (ΔCt = Ct _target gene_ − Ct _Gapdh_). The fold change was calculated as 2^−ΔΔCt^ (ΔΔCt = ΔCt _control_ − Ct _treatment_).

### 2.9. Ovariectomized Mouse Model

Seven-week-old female ddY mice (body weight, 20–23 g), including ovariectomized (OVX, *n* = 24) and sham-operated (sham, *n* = 6), were purchased from the Shizuoka Laboratory Center, Inc. (Hamamatsu, Japan). After surgery, the mice were acclimated for 1 week before scopolin treatment and were divided into five groups comprising six mice each: 1) Sham, 2) OVX control, 3) OVX treated with 10 mg.kg^−1^day^−1^ of SrCl_2_ as a positive control, 4) OVX treated with 20 mg.kg^−1^day^−1^ of scopolin, and 5) OVX treated with 40 mg.kg^−1^day^−1^ of scopolin. The mice were maintained on a diet of Formula-M07 (5.0 g/day; Feedlab Co., Ltd., Hanam, Korea) and tap water (15 mL/day). All the mice were kept in clear individual plastic cages under illumination (12-h light/dark cycle), temperature (23 ± 2 °C), and humidity (55% ± 5%). The mice were orally treated with the liquid form of scopolin alone (20 and 40 mg.kg^−1^day^−1^) by gavage for 12 weeks. The animal research protocol was approved by the Animal Care and Use Committee of the Ajou University School of Medicine, and all experiments were conducted in accordance with the institutional guidelines established by the Committee (AMC-133).

### 2.10. Measurement of Bone Mineral Density (BMD)

At the end of the animal experiment, the mice were anesthetized with tiletamine/zolazepam (Zoletil; Virbac Laboratories, Carros, France), and placed carefully on a specimen tray in the same position for measurements of right femur BMD using a PIXI-mus bone densitometer with the on-board PIXI-mus software (GE Lunar, Madison, WI, USA).

### 2.11. Microcomputed Tomography (Micro-CT) and Single-Photon Emission Computerized Tomography Scan

Transverse micro-CT images of the samples were scanned using a micro-CT scanner (INVEON, SIEMENS, Munich, Germany). Reconstruction was performed using the Inveon Research Workplace and COBRA_Exxim (Siemens, Munich, Germany). Two-dimensional (2D) axial and three-dimensional (3D) images were reconstructed for qualitative and quantitative analyses. These analyses provided information regarding the main histomorphometric parameters, including percentage bone volume (bone volume [BV]/tissue volume [TV], %), trabecular number (Tb.N, 1/mm), thickness (Tb.Th, l mm), and separation (Tb.Sp, l mm). Axial images were displayed using the Inveon Research Workplace (Siemens, Munich, Germany) for the region of interest (ROI) measurement and analyses. Axial reformats were performed to allow slice-by-slice manual tracing of the contours of the trabecular bone. A region 300 μm below the growth plate was analyzed for a cross-sectional study of the trabecular bone. For the single-photon emission computerized tomography (SPECT) scan, the mice were anesthetized with isoflurane/N_2_O/O_2_ inhalation anesthesia and injected with Tc-99m hydroxymethylene diphosphonate (HDP) (Mallinckrodt Medical B.V, Amsterdam, Netherlands) via the tail vein. Skeletal technetium (Tc-99m) HDP uptake on planar HDP bone scans of mice receiving intravenous treatments was plotted as a function of skeletal blood flow and osteogenic activity. In vivo planar images were obtained using an Inveon SPECT scanner (Siemens Preclinical Solutions, Malvern, PA, USA) equipped with a low-energy all-purpose collimator. The images were acquired until 100,000 total counts were measured per body.

### 2.12. Blood Sampling and Serum Level Measurements

The animals were euthanized, and fresh blood samples were collected from the left ventricle in heparinized syringes. Serum was immediately separated via centrifugation at 1200× *g* for 15 min at 4 °C and assayed for the measurement of OPG and RANKL using a Luminex multiplex assay (Merck Millipore, Burlington, MA, USA), according to the manufacturer’s instructions. The ratio of OPG to RANKL (OPG/RANKL) was calculated to explain the process of bone formation coupled with bone resorption.

### 2.13. Statistical Analysis

At least three biological and technical replicates were conducted during all experiments. The statistical package for the social sciences (SPSS 11.0 for Windows, SPSS Inc., Chicago, IL, USA) was used for statistical analyses. The results of bar graphs are expressed as the mean ± standard error of the mean. Differences between groups were assessed using Student’s *t*-test. A one-way analysis of variance was conducted for the comparisons of multiple groups, followed by Tukey’s honest significant difference post hoc test. *P*-values less than 0.05 (*p* < 0.05) were considered statistically significant.

## 3. Results

### 3.1. Screening for Cellular Proliferation and Differentiation in Osteoblastic MC3T3-E1 Cell Lines

**Figure 1:**
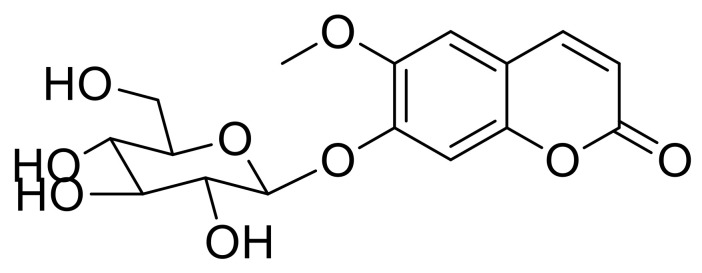
Chemical structure of scopolin.

Our previous study suggested that natural herbal medicine (LRC extract) prevented OVX-induced BMD loss in an osteoporotic mouse model by promoting the differentiation of osteoblast lineage cells [19]. In the present study, we screened the bioactive compound(s) fractionated from the LRC extract, which enhances bone formation (Figure S1). Alkaline phosphatase (ALP) is a glycoprotein located on the cell membrane of osteoblasts, and is a reliable and sensitive marker of bone metabolism; thus, we conducted an ALP assay to screen for bone formation-enhancing effects [22,23]. Consequently, a single bioactive compound was identified as scopolin using nuclear magnetic resonance (NMR) and mass spectrometry ((Figure 1 and Figure S2).

### 3.2. Scopolin Treatment Increased Osteoblast Differentiation and Mineralized Nodule Formation

**Figure 2:**
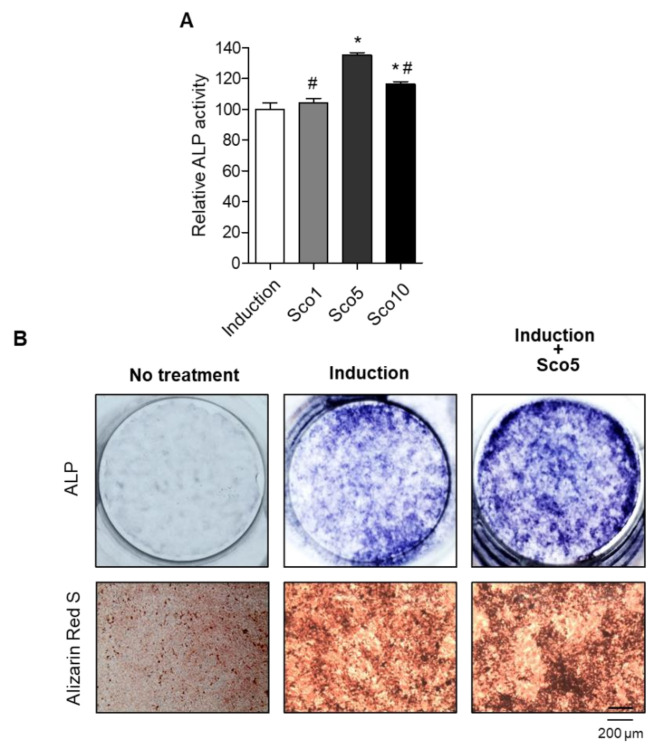
Scopolin treatment increased cellular differentiation and mineralized nodule formation in the MC3T3-E1 osteoblastic cell line. (**A**) The cells were cultured in three different concentrations of scopolin (1, 5, and 10 μM) for 3 days, and alkaline phosphatase (ALP) activity was measured. Measurements of ALP activity were repeated three times. (**B**) ALP and alizarin red S staining of cells treated with 5 μM scopolin. At least three replicate images were captured for each stained sample. Sco 1: scopoline 1 μM, Sco 5: scopoline 5 μM, Sco 10: scopoline 10 μM, Induction: Non-scopolin-treated cells. * *p* < 0.05 vs. Induction, ^#^ *p* < 0.05 vs. Sco5 (Tukey’s honest significant difference post hoc test, analysis of variance).

We investigated the specific anti-osteoporotic effects of scopolin on osteoblastic differentiation in the pre-osteoblastic MC3T3-E1 cell line. The cells were treated with three different concentrations of scopolin (1, 5, and 10 μM) for either 3 or 5 days (Figure S3). After incubation, cell viability and active bone formation were determined using WST and ALP assays, respectively. Scopolin treatment did not affect the proliferation of osteoblastic cells during the differentiation period (Figure S4). After 3 days of osteoblast differentiation induction, the greatest ALP activity was observed at a scopolin concentration of 5 μM (Figure 2A and Figure S3). Therefore, we used 5 μM scopolin for subsequent osteoblast differentiation experiments. In addition, treatment with scopolin increased ALP-positive staining compared to the non-scopolin-treated group (Figure 2B). To further confirm the effect of scopolin on calcium deposits in mineralized osteoblast cells, the bone-forming activity of scopolin was evaluated in MC3T3-E1 cells by measuring mineralized nodule formation, using alizarin red S staining [24,25]. After culturing MC3T3-E1 cells for 21 days, the scopolin-treated cells showed more alizarin red S-stained colonies than the non-scopolin-treated cells (Figure 2B).

### 3.3. Scopolin Treatment Increased the mRNA Expression of Osteoblastic Makers

**Figure 3:**
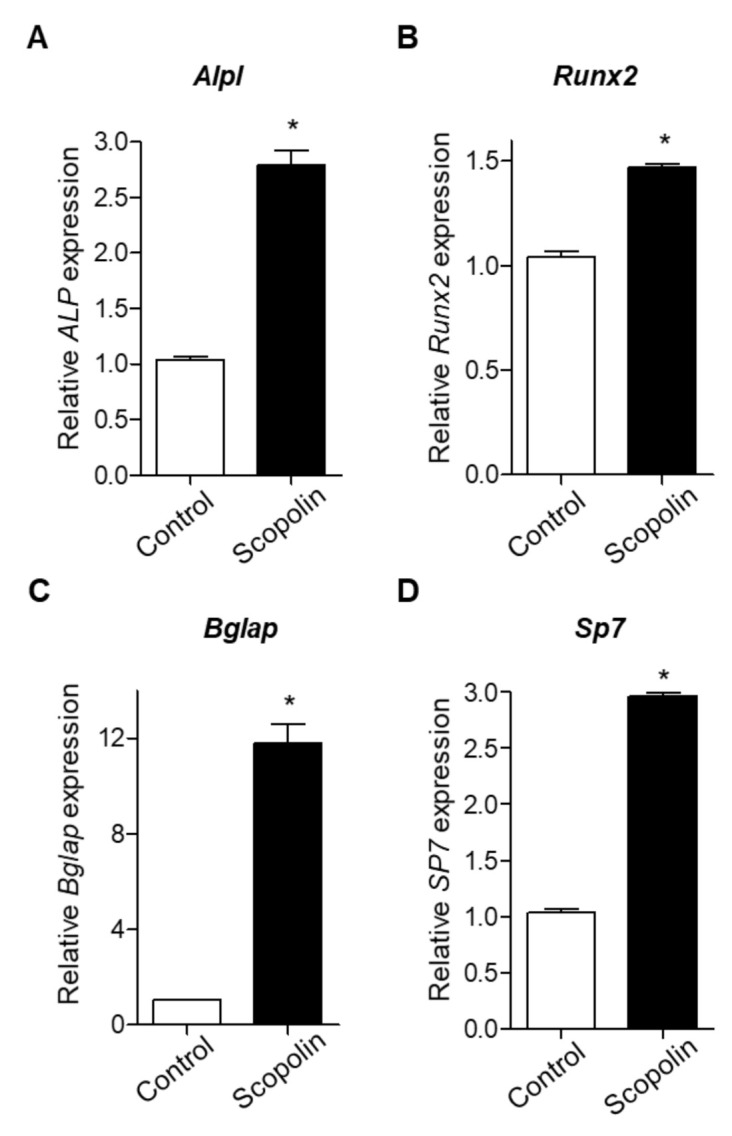
Scopolin treatment increased the mRNA levels of bone remodeling markers in MC3T3-E1 cells. The cells were treated with 5 μM scopolin. The mRNA levels of *Alpl* (alkaline phosphatase, ALP) (**A**), *Runx2* (runt-related transcription factor 2) (**B**), *Bglap* (bone gamma-carboxyglutamic acid-producing protein, osteocalcin) (**C**), and S*p7* (osterix) (**D**) were calculated quantitatively using reverse-transcription PCR (RT-PCR) with targeted gene-specific primers. Control: Non-scopolin-treated MC3T3-E1 cells. * *p* < 0.05 vs. Control. The measurements of mRNA expression levels were repeated three times.

Next, we examined the mRNA expression of bone remodeling markers, including *Alpl* (alkaline phosphatase, ALP), *Runx2* (runt-related transcription factor 2), *Bglap* (bone gamma-carboxyglutamic acid-producing protein, osteocalcin), and S*p7* (osterix), as previously suggested [26,27]. MC3T3-E1 cells were treated with 5 μM scopolin for 3 days. The mRNA expression of *Alpl*, *Runx2, Bglap*, and *Sp7* was significantly increased by scopolin treatment compared to the control group (Figure 3A–D). These results indicate that scopolin enhances osteoblast differentiation and mineralized nodule formation by upregulating bone remodeling markers (*Alpl*, *Runx2*, *Bglap*, and *Sp7*).

### 3.4. Scopolin Treatment Decreased Osteoclast Cellular differentiation

**Figure 4:**
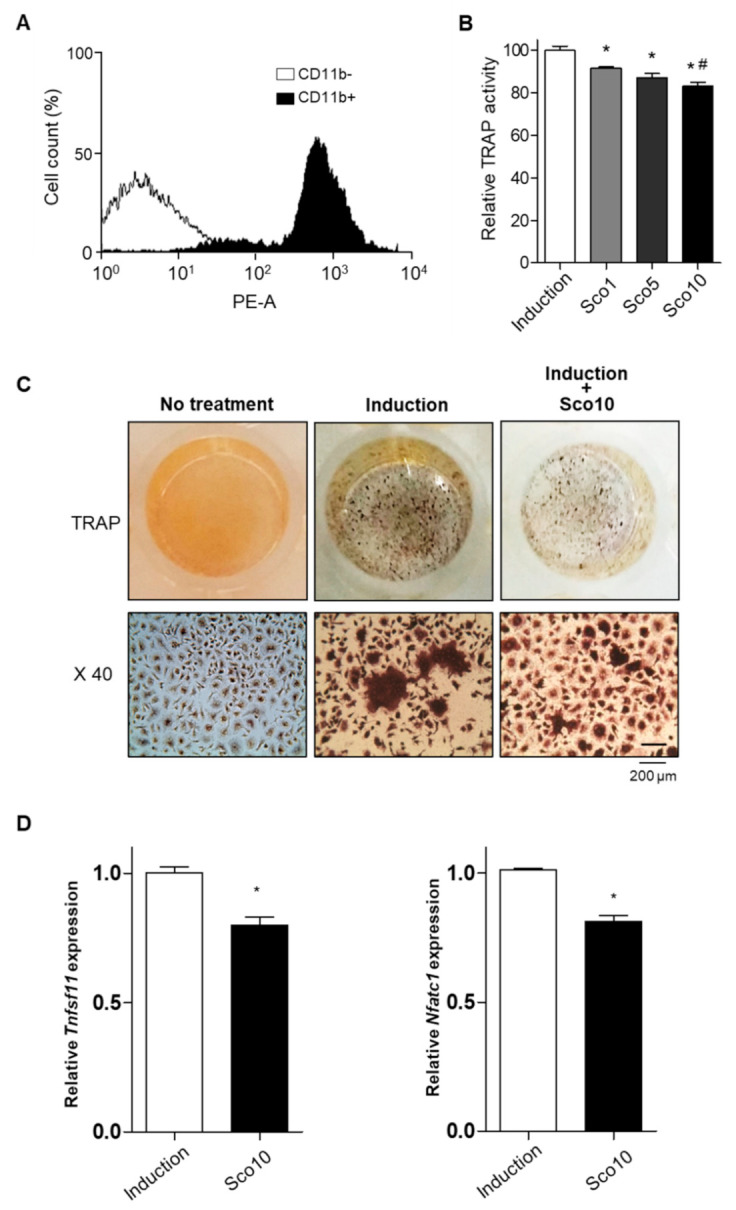
Scopolin treatment decreased the cellular differentiation of osteoclast cells isolated from bone marrow. (**A**) Monocytes successfully isolated from mouse bone marrow were identified by immunophenotypic analysis with a monocyte-specific surface positive marker (cluster of differentiation molecule 11B (CD11b) antibody). (**B**,**C**) Monocyte cells were cultured for 5 days and osteoclast differentiation was analyzed via (**B**) tartrate-resistant acid phosphatase (TRAP) activity assay and (**C**) TRAP staining. (**D**) The mRNA levels of osteoclast markers (tumor necrosis factor (ligand) superfamily, member 11 (*Tnfsf11*), and nuclear factor of activated T cells cytoplasmic 1 *(Nfatc1*)). X40: 40x magnification of TRAP staining. Induction: Non-scopolin-treated monocytes. * *p* < 0.05 vs. Induction, ^#^ *p* < 0.05 vs. Sco1 (Tukey’s honest significant difference post hoc test, analysis of variance). All the experiments were repeated three times.

Patients with osteoporosis, especially postmenopausal women, exhibit excessive bone resorption, resulting in an increased risk of fractures [28]. To examine the effects of scopolin on bone resorption, we investigated the cellular differentiation of monocytes (pre-osteoclastic cells) isolated from the bone marrow cells of seven-week-old mice. Monocytes were successfully isolated and detected using FACS immunophenotypic analysis and monocyte-specific surface markers (cluster of differentiation molecule 11B (CD11b) antibody) (Figure 4A). Scopolin treatment was not toxic to mouse bone marrow primary-cultured monocytes during the differentiation period. Osteoclast differentiation was measured with a tartrate-resistant acid phosphatase (TRAP) activity assay and TRAP staining after either 3 or 5 days of incubation (Figure S5A). Scopolin did not affect cell proliferation in monocytes (Figure S5B). Scopolin treatment decreased the cellular differentiation of osteoclasts in isolated mouse primary monocytes in a dose-dependent manner after 5 days of incubation (Figure 4B). TRAP staining of differentiated osteoclasts consistently showed reduced osteoclast differentiation after scopolin treatment for 5 days (Figure 4C). Tumor necrosis factor (ligand) superfamily, member 11 (*Tnfsf11*), and nuclear factor of activated T cells cytoplasmic 1 (*Nfatc1*) were measured to further confirm the effects of scopolin on the mRNA levels of osteoclast markers. Scopolin treatment significantly reduced the expression of osteoclast differentiation-induced genes (Figure 4D). These results suggest that scopolin treatment inhibited osteoclast differentiation by suppressing osteoclast-inducing factors.

### 3.5. Scopolin Treatment Increased Osteoblast Differentiation in an Isolated Monocyte/MC3T3-E1 Co-Culture System

**Figure 5:**
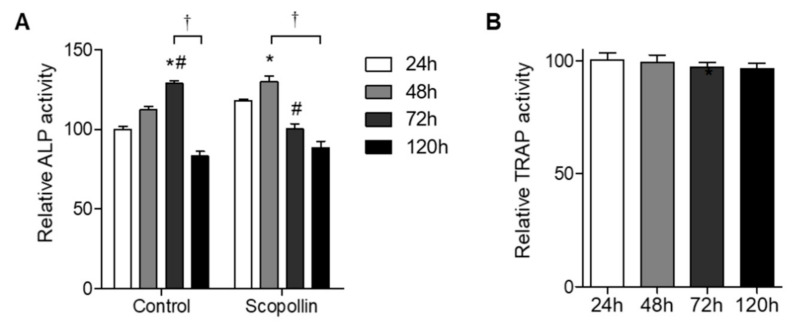
Scopolin treatment increased osteoblast differentiation in a co-culture of isolated monocytes and MC3T3-E1 osteoblastic cells. Isolated monocyte (4 × 10^4^ cells/well) and MC3T3-E1 cells (2 × 10^4^ cells/well) were treated with 5 μM of scopolin, and (**A**) alkaline phosphatase (ALP) and (**B**) tartrate-resistant acid phosphatase (TRAP) activity were measured at 24, 48, 72, and 120 h. At least three different experiments were performed. * *p* < 0.05 vs. 24 h, ^#^ *p* < 0.05 vs. 48 h, and ^†^ *p* < 0.05 vs. 120 h (Tukey’s honest significant difference post hoc test, analysis of variance).

Since bone remodeling under physiological conditions is regulated by the balance between bone resorption and bone formation, we tested the effect of scopolin on osteoblast and osteoclast differentiation in a co-culture system. Preosteoclast monocytes isolated from mouse bone marrow and preosteoblast MC3T3-E1 cells were co-cultured for 24, 48, 72, and 120 h, and ALP activity for osteoblast differentiation and TRAP activity for osteoclast differentiation were measured. Greater osteoblast differentiation was observed in the co-culture system of monocytes and MC3T3-E1 cells than in MC3T3-E1 cells alone (Figure S6). ALP activity in scopolin-treated cells peaked earlier at 48 h than in the control group at 72 h (Figure 5A), indicating that scopoline enhanced osteoblast differentiation. However, scopolin treatment did not change osteoclast differentiation in the co-culture system (Figure 5B). These results suggest that scopolin promoted osteoblast differentiation without affecting osteoclast differentiation in a co-culture of monocytes and MC3T3-E1 cells.

### 3.6. Scopolin Treatment Prevented BMD Loss in OVX Mice

**Figure 6:**
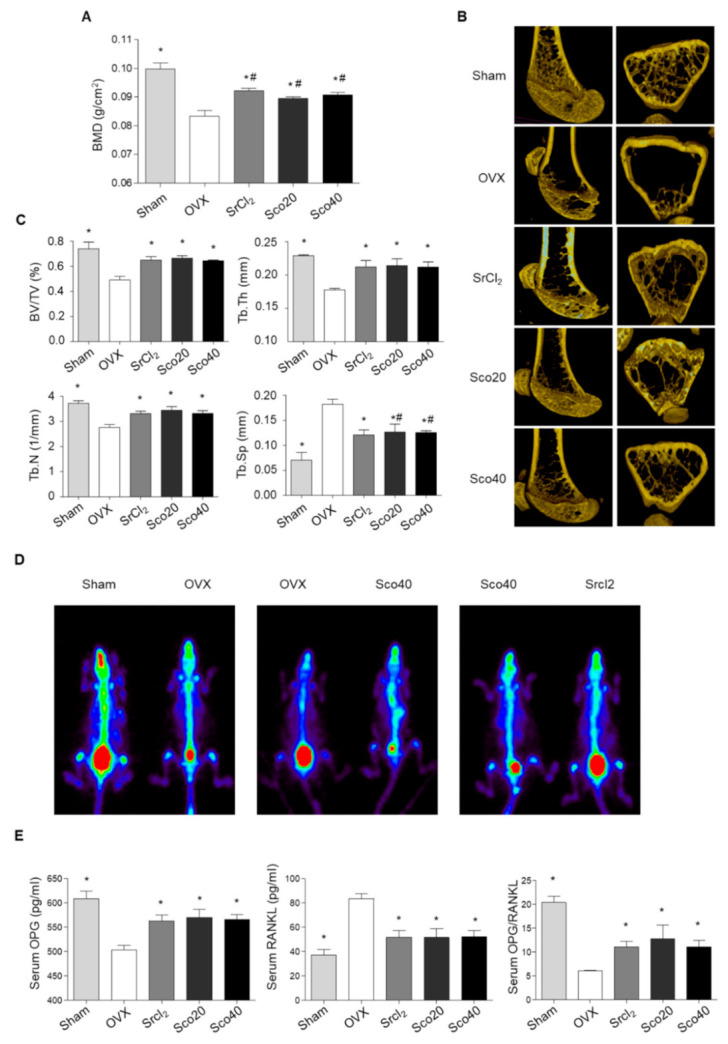
Scopolin treatment prevented ovariectomized (OVX)-induced bone loss in mice. The OVX mice were treated with either SrCl_2_ (10 mg.kg^−1^day^−1^) or scopolin (20 or 40 mg.kg^−1^day^−1^) for 12 weeks. Sham: Sham-operated, OVX: Non-scopolin-treated mice. (**A**) The right femur bone mineral density (BMD) was measured using a PIXI-mus bone densitometer at 12 weeks. (**B**) Transverse micro-CT images of the right femur and (**C**) bone volume fraction (BV/TV), trabecular thickness (Tb.Th), trabecular number (Tb.N), and trabecular spacing (Tb.Sp) were analyzed. (**D**) Technetium Tc-99m hydroxymethylene diphosphonate (HDP) bone scan was taken with an Inveon single-photon emission computerized tomography (SPECT) scanner. (**E**) Osteoprotegerin (OPG) and of receptor activator of nuclear factor-κB (NF-κB) ligand (RANKL) were measured using enzyme-linked immunosorbent assay (ELISA) and the ratio of OPG/RANKL was calculated. * *p* < 0.05 vs. OVX control and ^#^ *p* < 0.05 vs. OVX control (Tukey’s honest significant difference post hoc test, analysis of variance). All experiments were repeated three times, except the measurement of skeletal technetium (Tc-99m) HDP uptake on planar HDP bone, which was performed once.

Based on the results of osteoblast and osteoclast differentiation, we investigated the anti-osteoporotic effects of scopolin in an OVX mouse model. The OVX mouse model is a commonly used surrogate for postmenopausal humans and is characterized by reduced bone mass and quality with significantly decreased bone mineral density (BMD) and bone mineral content (BMC) [29]. BMD was measured on the last day of treatment using a PIXI-mus bone densitometer. Transverse micro-CT images of the right femur were obtained, and bone properties including bone volume (BV/TV), trabecular thickness (Tb.Th), number (Tb.N), and spacing (Tb.Sp) were analyzed. As expected, OVX mice showed significant osteoporotic trabecular bone loss compared to the sham mice (Figure 6A). However, SrCl_2_ treatment for 12 weeks (positive control) restored BMD in the right femur bone with improved trabecular bone structural properties, including BV/TV, Tb.Th, Tb.N, and Tb.Sp (Figure 6A–C). The BMD of the right femur increased significantly in the groups treated with 20 and 40 mg.kg^−1^day^−1^ scopolin for 12 weeks, compared to that of the OVX control group (Figure 6A). In addition, scopolin treatment prevented OVX-induced trabecular bone loss and significantly improved BV/TV, Tb.Th, Tb.N, and Tb.Sp compared to the OVX control group (Figure 6B,C). For comparison, paired mice were scanned in the same panel. A higher level of radioactivity was detected in the sham and scopolin-treated groups compared to the OVX group, indicating enhanced osteogenesis in osteoporotic mice (Figure 6D).

We collected blood samples and measured serum levels of bone metabolic markers, osteoprotegerin (OPG), and RANKL, using enzyme-linked immunosorbent assay (ELISA). SrCl_2_ increased OPG and decreased RANKL serum levels compared to non-treated OVX mice. Scopolin treatment also significantly increased serum OPG levels and decreased serum RANKL levels (Figure 6E). Thus, the OPG/RANKL ratio increased significantly following scopolin treatment (Figure 6E).

## 4. Discussion

In this study, we isolated and identified a single bioactive compound, scopolin, from the fractionation of LRC. Scopolin treatment enhanced osteoblast differentiation and mineralization in MC3T3-E1 cells by upregulating the mRNA expression of *Alpl*, *Runx2, Bglap*, and *sp7*. In contrast, decreased osteoclast differentiation in primary cultured mouse monocytes from bone marrow was observed after scopolin treatment. Consistently, treatment of a monocyte and MC3T3-E1 co-culture system with scopolin resulted in enhanced osteoblast differentiation, and scopolin treatment prevented OVX-induced bone loss and osteoclastogenesis in an OVX animal model.

A previous study demonstrated that LRC might be an alternative herbal drug for effective osteoporosis treatment in vivo and in vitro and suggested 13 major compounds identified in LRC extract [19]. In addition, a study reported that the bioactive component Kukoamin B, isolated from LRC, showed anti-osteoporotic effects [30]. We found that another single bioactive compound, scopolin, promoted bone formation in the MC3T3-E1 cell line. New bone formation is the principal function of osteoblasts; therefore, osteoblast differentiation can promote bone formation [31]. We observed that scopolin showed inductive effects on osteoblast differentiation without cell proliferation, indicating an enhanced effect on new bone formation without a toxic effect on cell growth. The bone matrix is mineralized by osteoblasts, resulting in the stimulation of calcium production with the induction of bone mineralization [32]. Alizarin red S is a reliable histochemical stain for measuring calcium deposits in mineralized osteoblast cells [25]. Positive alizarin red S staining demonstrates the presence of calcium phosphate and osteoblast mineralization, indicating successful in vitro bone formation [33]. The mineralized matrix and nodule formation were enhanced by scopolin treatment. Interestingly, scopolin treatment appeared to stimulate osteoblast differentiation by activating osteoblastic-inducing genes, including *Alpl*, *Runx2*, *Bglap*, and *sp*7, which play crucial roles in the mineralization of newly formed bone.

A previous study demonstrated that scopolin exerted suppressive effects on the differentiation of pro-osteoclastic RAW 264.7 cells by inhibiting RANKL-induced osteoclast differentiation [34]. Similarly, our study found that scopolin treatment reduced osteoclast differentiation in monocytes isolated from mouse bone marrow. In addition, treatment of a co-culture system of monocyte and MC3T3-E1 cells with scopolin enhanced osteoblast differentiation but did not affect osteoclast differentiation. This is probably because differentiated osteoblasts promote the release of M-CSF and RANKL in culture media. These results suggest that scopolin promotes osteoblast differentiation during bone remodeling.

We also found that scopolin treatment inhibited OVX-induced bone loss in mice. We selected SrCl_2_ as a positive anti-osteoporotic agent because a previous report suggested the protective effects of SrCl_2_ against bone loss [35]. Scopolin treatment prevented osteoporotic BMD loss in OVX mice and significantly improved BV/TV, Tb.Th, Tb.N, and Tb.Sp based on the analysis of 2D and 3D micro-CT images of the femur. Consistent with the protective effects of scopolin on bone loss, we also found that scopolin affects bone metabolic markers (RANKL and OPG). Many studies have demonstrated that the OPG/RANKL signaling system is a key mediator of osteoclast maturation and bone formation during bone remodeling [36]. RANKL is a key mediator of bone resorption, and OPG is well known as an inhibitor of osteoclastogenesis and a decoy receptor for RANKL, preventing its binding to the receptor activator of nuclear factor-kappa B [37,38]. OVX rats have increased serum levels of RANKL [37]. However, inhibition of RANKL by OPG secretion causes decreased bone loss in estrogen-ablated animal models through the suppression of bone resorption [36]. Thus, scopolin treatment protects against unbalanced OPG and RANKL serum levels in OVX mice, thereby promoting bone formation with preserved trabecular architecture. Our results suggest that scopolin treatment prevented OVX-induced elevation of RANKL and decreased OPG serum levels, indicating the inhibition of osteoclastogenesis in osteoporotic OVX mice.

## 5. Conclusions

We investigated the effect of scopolin treatment on OVX-induced bone loss in an osteoporotic animal model. Our data suggested that scopolin treatment enhanced osteoblastic differentiation by upregulating osteoblastic-inducing genes. Consequently, the activation of osteoblastic signaling induced by scopolin treatment prevented OVX-induced bone loss in mice. Our observations suggest that scopolin might be a potential alternative herbal medicine for the prevention of bone loss.

## Supplementary Materials

The following are available online at https://www.mdpi.com/2072-6643/12/11/3565/s1, Figure S1: Fractionation and isolation of the bioactive component isolated from LRC, Figure S2: ^1^H-NMR (a), ^13^C-NMR (b), and mass (c) spectrum of scopolin; Figure S3: Scopolin increased alkaline phosphatase (ALP) activity after 3 and 5 days of incubation. The cells were cultured with three different concentrations of scopolin (1, 5, and 10 μM), and alkaline phosphatase (ALP) activity was measured. * *p* < 0.05 vs. control, Figure S4: Scopolin did not affect the proliferation of osteoblastic cells. The cells were cultured with three different concentrations of scopolin (1, 5, and 10 μM) for 5 days, and cell viability was measured. Figure S5: Scopolin reduced osteoclastic differentiation in monocytes isolated from the bone marrow. The monocyte cells were cultured for 3 and 5 days in the presence of 30 ng/mL M-CSF and 50 ng/mL RANKL (induction) or in the presence of M-CSF and RANKL with 1, 5, or 10 μM of scopolin. * *p* < 0.05 vs. control, Figure S6: Co-culture of isolated monocytes with MC3T3-E1 osteoblastic cell line cells increased osteoblast differentiation compared to a single-culture of MC3T3-E1 cells. The isolated monocytes and MC3T3-E1 cells were co-cultured for 48 h and ALP activity was measured to determine osteoblast differentiation. * *p* < 0.05. vs. MC3T3-E1.
