# Supplementary material for: Scopolin Attenuates Osteoporotic Bone Loss in Ovariectomized Mice
Source: Nutrients. 2020 Nov 20;12(11):3565. doi: 10.3390/nu12113565 (PMC7699886; doi:10.3390/nu12113565)
Supplement: Supplementary file 1 [file nutrients-12-03565-s001.pdf]

Scopolin treatment prevents osteoporotic bone loss in ovariectomized mice

Eunkuk Park, Jeonghyun Kim, Hyun-Seok Jin, Chun Whan Choi, Tae Hyun Choi,

Sangho Choi, Dam Huh, and Seon-Yong Jeong

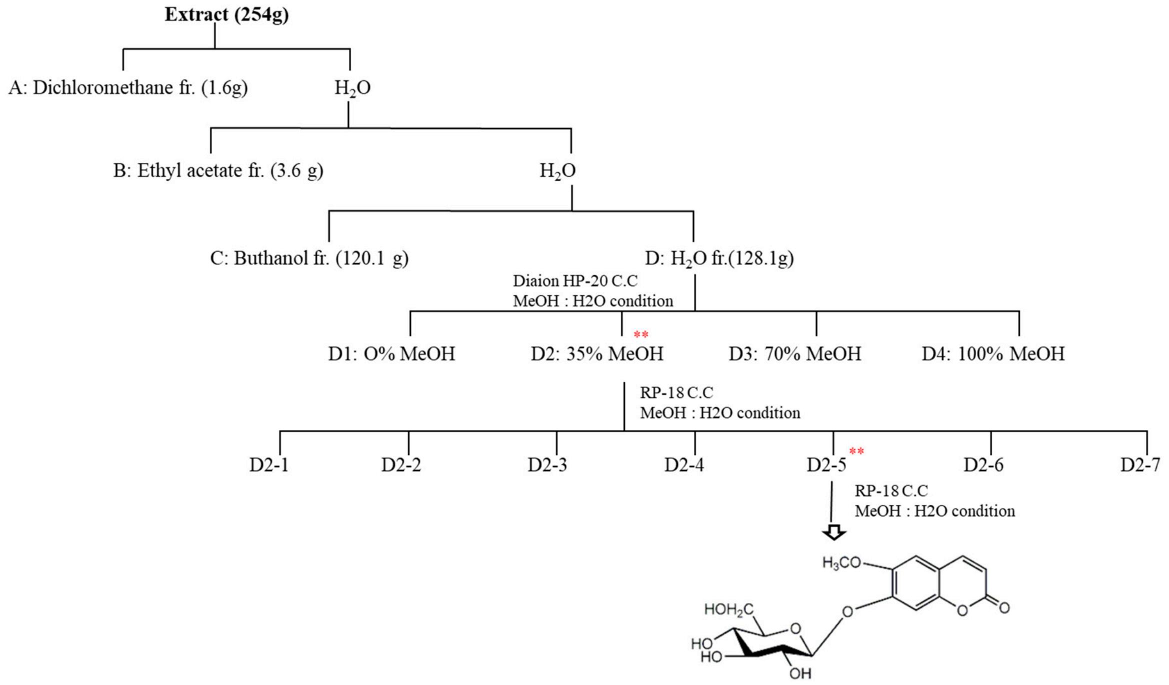

Figure S1. Fractionation and isolation of the bioactive component isolated from LRC

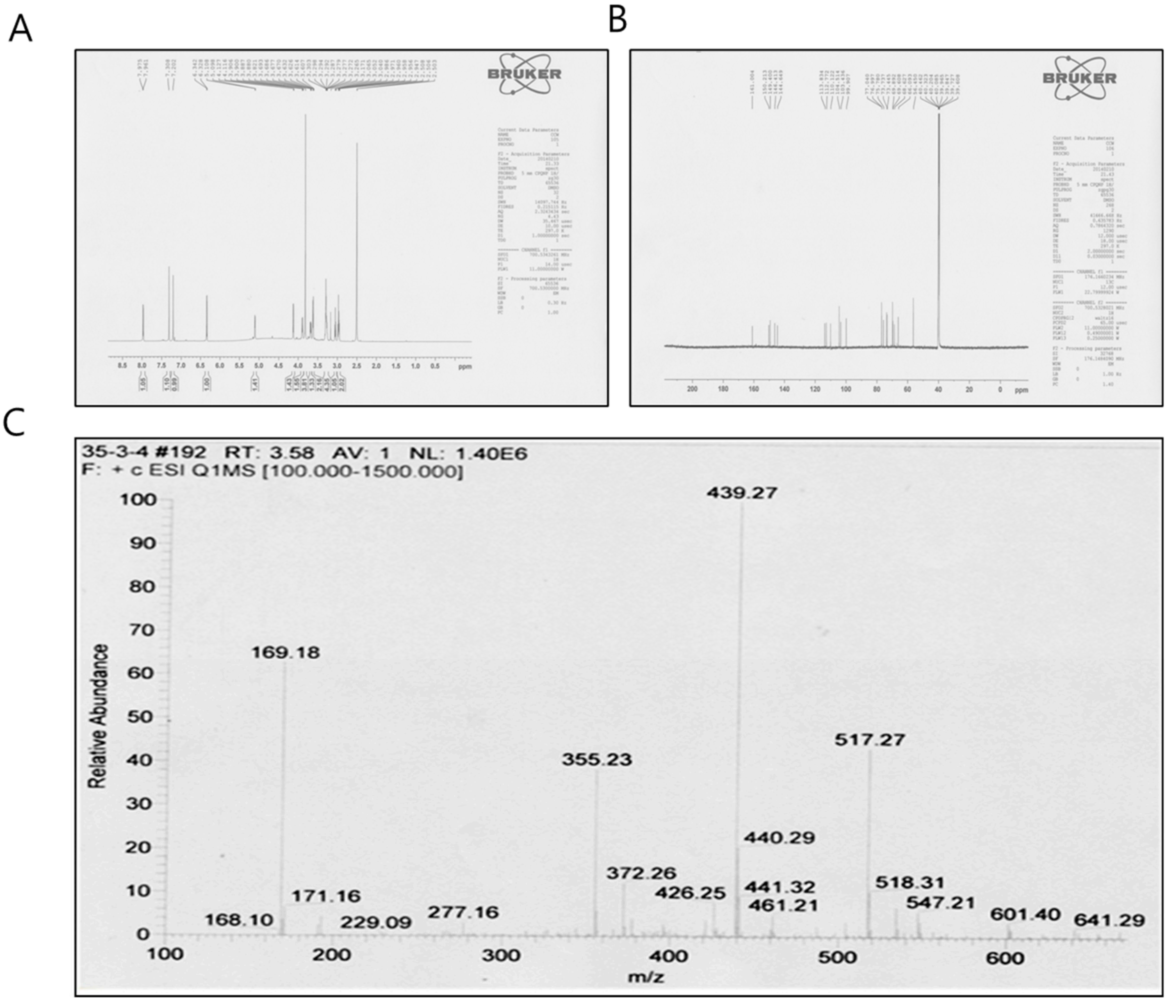

Figure S2. ^{1}H-NMR (A), ^{13}C-NMR (B), and mass (C) spectrum of scopolin.

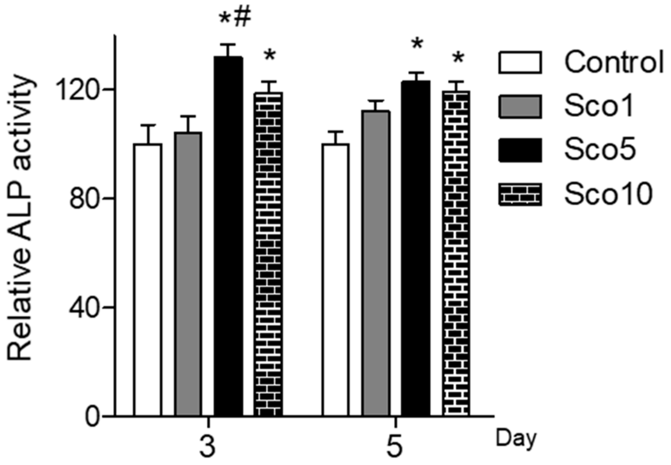

Figure S3. Scopolin increased alkaline phosphatase (ALP) activity after 3 and 5 days of incubation. The cells were cultured with three different concentrations of scopolin (1, 5 and 10 μM), and alkaline phosphatase (ALP) activity was measured. *p < 0.05 vs. control, #p < 0.05 vs. Sco1.

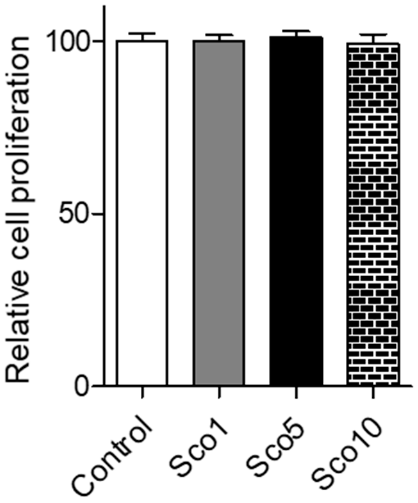

Figure S4. Scopolin did not affect proliferation of osteoblastic cells. The cells were cultured with three different concentrations of scopolin (1, 5 and 10 μM) for 5 days, and the cell viability was measured.

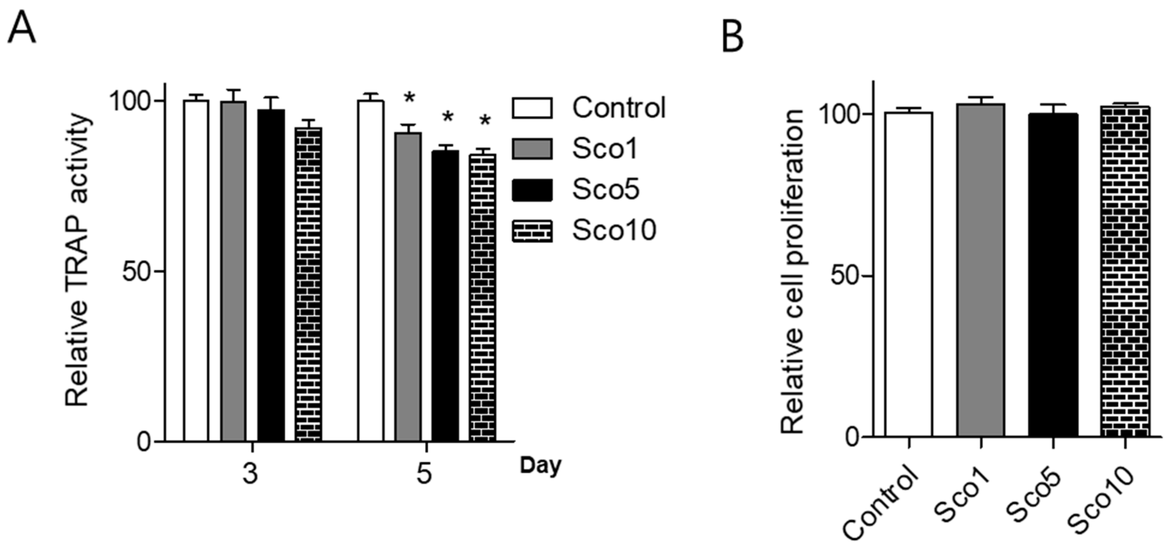

Figure S5. Scopolin reduced osteoclastic differentiation (A) and did not affect cell proliferation (B) in monocytes isolated from bone marrow. The monocyte cells were cultured for 3 and 5 days in the presence of 30 ng/ml of M-CSF and 50 ng/ml of RANKL (induction) or in the presence of M-CSF and RANKL with 1, 5, or 10 μM of scopolin. *$p < 0.05$ vs. control.

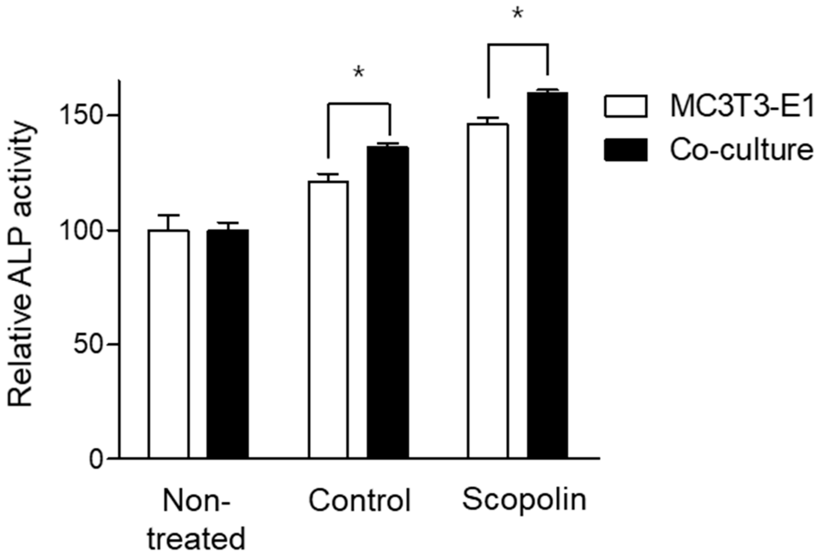

Figure S6. Co-culture of isolated monocytes with MC3T3-E1 osteoblastic cell line cells increased osteoblast differentiation compared to a single-culture of MC3T3-E1. The isolated monocyte and MC3T3-E1 cells were co-cultured for 48 hours and ALP activity was measured to determine osteoblast differentiation. *: $p < 0.05$. vs. MC3T3-E1.
